# Effects of rotation and *Bacillus* on the changes of continuous cropping soil fungal communities in American ginseng

**DOI:** 10.1007/s11274-023-03807-w

**Published:** 2023-10-24

**Authors:** Fengan Jia, Fan Chang, Min Guan, Qingan Jia, Yan Sun, Zhi Li

**Affiliations:** 1 Shaanxi Institute of Microbiology, Xi’an, 710043 China; 2 College of Life Science, Shaanxi Normal University, Xi’an, 710062 China; 3 Shaanxi Agricultural Machinery Research Institute, Xianyang, 712000 China; 4 Institute of Medical Research, Northwestern Polytechnical University, Xi’an, 710072 China

**Keywords:** American ginseng, Fungal community, Dynamic changes, Continuous cropping obstacle, Crop rotation

## Abstract

The continuous cropping obstacle is the main factor in leading to difficulty in American ginseng replanting. The dormant microbiota in the soil may be the cause of American ginseng disease and eventually caused continuous cropping obstacles, but there are few studies on the dynamic changes of soil microenvironment after American ginseng planting. In this study, we tracked short-term variation in physicochemical properties, enzyme activities, and fungal communities over time-series in soils with continuous cropping obstacle under crop rotation and probiotic *Bacillus* treatments. Furthermore, we examined the relationships between the important fungal compositions and the soil properties. The results showed that sucrase, cellulase, urease and acid phosphatase activities were significantly increased, while catalase and dehydrogenase were decreased with treatments time. Rotation treatment significantly affected the diversity, dissimilarity degree and species distribution of soil fungal community with continuous cropping obstacle over a short-term. Moreover, beneficial fungal biomarkers such as *Cladorrhinum*, *Oidiodendron*, and *Mariannaea* were accumulated at 48 h under rotation treatments. Almost all fungal biomarkers were negatively correlated with hydrolases and positively correlated with oxidoreductases and acid phosphatase under crop rotation treatments. This study suggested that compared to probiotic *Bacillus*, crop rotation can significantly affect soil fungal community structure, especially the enrichment of specific potentially beneficial fungal species. Our findings provide a scientific basis for understanding the dynamic changes of fungal communities and soil properties with continuous cropping obstacle of American ginseng in initial stage of soil improvement.

## Introduction

The cropping obstacle, characterized by reduced crop yield and quality resulting from prolonged monoculture continuous cropping, has been observed in both annual and perennial crops (Tan et al. 2021). After harvest, replanting of traditional Chinese medicine will also cause soil continuous cropping obstacles, resulting in slow growth, increased incidence of soil-borne diseases, and decreased yields (Tagele et al. 2023; Wang et al. 2023b). The continuous cropping obstacle of traditional Chinese medicine has always been a bottleneck problem in the development of traditional Chinese medicine, especially in the cultivation of American ginseng (*Panax quinquefolius* L.) (Xiao et al. 2016). Due to its slow growth, American ginseng usually requires 4 years of growth to achieve optimal biomass and active component content (Zhang et al. 2023b). However, the soil cultivated the American ginseng for one season can lead to a decline in both yield and quality on the same land for 10 years or more (Li et al. 2020). Although American ginseng has adopted “two-year land-changing planting” transplanting mode in northwest China, soil continuous cropping obstacle is still unavoidable and seriously restricts the local American ginseng cultivation (Chang et al. 2022). The cropping obstacle may include numerous biotic and abiotic factors, e.g., changes in soil physicochemical properties and enzyme activities (Chung et al. 2017; Wei et al. 2018; Zhang et al. 2020a). enriched soil-borne pathogenic microorganisms (Liu et al. 2021), and allelopathic effects of plants (Zhang et al. 2023c). Among them, soil microbiota are the main driving factor for soil nutrient cycling and transformation (Jiao et al. 2019a).

The establishment of the soil microbiota is a dynamic process. In particular, the initial microbial community can become more specific by selection for plant growth or different treatments (Bulgarelli et al. 2013; Tkacz and Poole 2015). In the studies of *Panax ginseng*, the increase of in cultivation years significantly changed the diversity of soil microbial communities and led to the accumulation of soil pathogenic fungi (Tong et al. 2021). Crop rotation could significantly affect the structure and composition of American ginseng continuous cropping soil microbial community, which were influenced by the alteration of soil properties (Liu et al. 2021). Our previous research also found the microbiota in soil and rhizosphere microhabitat had a tendency of gradual differentiation and specific enrichment during the growth process, especially in the first year of American ginseng cultivation (Chang et al. 2022). Fungal community is an essential component of soil microbiome which play an important role in material circulation, energy transfer, as well as inhibition or prevalence of soil-borne diseases (Li et al. 2022). Some studies have shown that the dynamic shifts of the fungal community with the cropping process of American ginseng could potentially change the soil composition, and the enrichment of potential pathogenic fungi may change the rhizosphere secretions, collectively impacting the soil environment and leading to the occurrence of continuous cropping obstacles (Bi et al. 2023; Zhang et al. 2023b). However, these studies only focused on specific time points during the growth of American ginseng or alterations in soil and plants pre- and post-intervention.

Crop rotation and *Bacillus amyloliquefaciens* biocontrol agent have been demonstrated to effectively mitigate the replanting difficulties and soil diseases development caused by soil continuous cropping obstacles (Li et al. 2022; Wang et al. 2023a). However, the reasons for the sustained alleviation of soil continuous cropping obstacles after initial intervention have not been fully explained. We hypothesized that the continuous cropping obstacles might be related to the dormant fungal community in the soil, and different soil improvement methods could regulate the structure and composition of the fungal community in the initial stage. Time series analysis has proven to be an effective strategy for exploring the dynamics in the structure and composition of soil microbiota and has been widely adopted in rice and *Arabidopsis* studies (Lundberg et al. 2012; Edwards et al. 2018). In this study, time-series trials were performed to track the dynamic shifts of fungal communities in American ginseng continuous cropping obstacle soil. In addition, soil properties and enzyme activities were detected in a correspondence soil sample to reveal their correlation with fungal communities and cropping obstacle. We explored important fungal compositions over time-series, revealing relationships between the fungal biomarker taxa and soil nutrients. The results obtained from this study will be valuable for gaining insight into the dynamic regulation of soil microecology under different soil interventions, which can provide reference for the effective improvement of soil with continuous cropping obstacles.

## Materials and methods

### Experimental design and collection of soil samples

The experiment was conducted in Liuba village, Shaanxi province, northwest China (latitude 33°40′N, longitude 106°52′E). The American ginseng was cultivated on Newly cultivated land. Organic fertilizer (4.0 kg m^− 2^) was applied as the base fertilizer before the American ginseng cultivation and subsequently top-dressed (4.0 kg m^− 2^) annually in March (Chang et al. 2022). The cultivation followed the standard operating procedures of Good Agricultural Practice (GAP) (Zhang et al. 2010). We collected the topsoil from 0 to 30 cm depth, which had caused continuous cropping obstacles after four years of American ginseng cultivation. The soil was transported back to the laboratory as soon as possible for crop rotation and probiotic treatments. The pot experiment was conducted and divided into 5 groups of 3 biological replicates as follows: watering only (NR_W); probiotic *Bacillus* agents only (NR_P); rotation and watering (R_W); rotation and probiotic *Bacillus* agents (R_P); and initial untreated soil control (CK).

*Bacillus amyloliquefaciens* JK1 (NBRC 15,535) and JK2 (BCRC 11,601) isolated from the previous study with the ability to secrete lipopeptide and siderophore (Wang et al. 2016) were used as probiotics treatments. The preparation of the probiotic agents was as follows: The activated JK1 and JK2 culture was inoculated into fresh nutrient agar medium (NA, QingDao Hopebio Technology Co., Ltd., Qingdao, China) respectively, to a final concentration of 10^10^-10^11^ CFU/mL as the probiotic solution. *Bacillus* solutions were diluted with 4 L sterile water to a final concentration of 10^7^-10^8^ CFU/mL and irrigated potting soil to reach 85% soil moisture. Eventually the initial soil sample contained about 10^3^-10^4^*Bacillus* per gram (dry weight) of soil. The watering treatment groups used the same volume of sterile water for the first time. After that, all treatment groups added the same amount of sterile water every 3 to 5 days to maintain the soil moisture of 60–70% until the end of the experiment.

The rotation treatment method was as follows: *Brassica chinensis* L., which is often rotated locally, was planted with 50 to 60 seeds per pot and the experiment period was 35 days. Before rotation, the seeds were soaked in 70% (v/v) ethanol for 3 min, sterile water washed seeds 3 times, and soaked in 5% sodium hypochlorite for 3 min, finally, washed 3 times with sterile distilled water to complete the surface sterilized.

Soil samples were collected at 48 h, 3d, 7d, 14d, 21d, 28d and 35d respectively, according to the plant growth process and the possible colonization time of the fungi. In total, 31 samples were collected from the soil under four different treatments (3 CK samples and 28 treatments samples consisted of 4 treatments, 7 time points). Each soil sample was thoroughly mixed and evenly divided into an average of 9 replicates and processed immediately. Three replicates were air-dried in a soil drying chamber, and the physicochemical components of the soil and total phenolic acids were determined. Another three replicates were stored in a refrigerator at 4 ℃, and soil enzyme activity was detected as soon as possible. The final three replicates were frozen with liquid nitrogen for 3 times and stored at -80 ℃ for high-throughput sequencing analysis.

### Soil properties and soil enzyme activity

The chemical and physical properties of soil samples were measured. Soil organic matter (OM) was determined using dichromate wet combustion according to a previously established method (De Falco et al. 2004). Soil total nitrogen (TN) and soil available nitrogen (AN) content, total phosphorus and available phosphorus (AP) content, and total potassium (TK) and available potassium (AK) content were determined as previously described (Xu et al. 2006; Chang et al. 2022). The soil pH and electrical conductivity (EC) were measured in a 1:5 aqueous soil-water suspension using a pH meter (PB-10, Sartorius, Germany) and conductometer (FE30, Mettler Toledo, Switzerland), respectively. The total phenolic content of the soil samples (Totalphenol, mg protocatechuic acid/100 g) was determined with Folin–Ciocalteu method at the wavelength of 765 nm (Singleton et al. 1999).

The activities of sucrase and cellulase were measured by 3,5-dinitrosalicylic acid colorimetry at a wavelength of 540 nm and expressed as 1 mg of reducing glucose production per gram of soil sample in 24 h. Urease activity was determined by indophenol colorimetry at a wavelength of 578 nm and expressed as mass (mg) of NH_3_-N in 1 g of soil after 24 h. Catalase activity was determined by potassium permanganate titration and expressed as potassium permanganate volume (mL) per gram of soil consumed in 1 h. Dehydrogenase activity was detected by 2, 3, 5-triphenyltetrazolium chloride (TTC) reduction method at a wavelength of 485 nm and expressed by the mass (µg) of 1, 3, 5-triphenylformazan (TPF) in 1 g of soil after 24 h. Acid phosphatase activity was determined by phenyl disodium phosphate colorimetry and measured at a wave length of 578 nm. Acetic acid buffer (pH = 5.0 ~ 5.4) was added to the acid phosphatase activity test (Guan 1986). All soil samples from each treatment with three replicates were tested.

### DNA extraction, PCR amplification, and sequencing

Total DNA was extracted from the samples using the FastDNA SPIN Kit according to the manufacturer’s protocol (MP Biomedicals, USA). Nuclear-free water was used as a blank. The total DNA was eluted in 50 µL of Elution buffer and stored at -80 °C until PCR by LC-Bio Co., Ltd.

Fungal internal transcribed spacer 2 (ITS2) region was amplified using primers ITS1FI2 (5′-GTGARTCATCGAATCTTTG-3′) and ITS2 (5′-TCCTCCGCTTATTGATATGC-3′) with specific barcodes per sample and sequencing universal primers attached to the 5’ ends of the primers (Karlsson et al. 2014). PCR amplification was conducted in a total volume of 25 µL reaction mixture containing 25 ng of template DNA, 12.5 µL PCR Premix, 2.5 µL of each primer, and PCR-grade water for volume adjustment. The PCR conditions for amplifying the ITS fragments consisted of an initial denaturation at 98 ℃ for 30 s, followed by 32 cycles of denaturation at 98 ℃ for 10 s, annealing at 54 ℃ for 30 s, and extension at 72 ℃ for 45 s. Finally, a final extension was performed at 72 ℃ for 10 min. The PCR products were confirmed by electrophoresis on a 2% agarose gel. Ultrapure water was used as a negative control during the DNA extraction process to eliminate the possibility of false-positive PCR results. The PCR products were purifyied using AMPure XT beads (Beckman Coulter Genomics, USA) and quantified with Qubit (Invitrogen, USA). Subsequently, the amplicon pools were prepared for sequencing and the size and quantity of the amplicon library were assessed on Agilent 2100 Bioanalyzer (Agilent, USA) and with the Library Quantification Kit for Illumina (Kapa Biosciences, USA), respectively. The libraries were sequenced on NovaSeq PE250 platform. The raw data from 31 samples were available from the Sequence Read Archive (SRA) under the accession number: PRJNA954137.

### Data analyses

The Usearch10 (Edgar 2010) analysis pipelines were used to evaluate the data. Following barcode assignment and primer sequence removal, forward and reverse reads were merged and assigned to respective samples. Quality filtering was performed with the criteria of no ambiguous bases and an expected errors per base rate > 0.01. The sequences were dereplicated, and singletons with a minimum unique size of less than 8 were removed. Then, the exact sequence variants algorithm (Knight et al. 2018; Edgar 2018) (Unoise3) was employed to cluster the sequences into amplicon sequence variants (ASVs), while simultaneously removing chimeric sequences. Sequences were clustered using the clustering program against the UNITE fungal ITS database (Bengtsson-Palme et al. 2013) and taxonomical assignment was performed with all ASVs at a confidence threshold of 0.8.

All statistical analyses were conducted using R software version 4.1.2. The alpha-diversity was assessed through the Richness, Shannon, and Pielou indices with the “vegan” package (version 2.5-6, https://CRAN.R-project.org/package=vegan) (Kembel et al. 2010). Multiple comparisons among groups were performed using one-way analysis of variance (ANOVA) followed by Tukey’s post-hoc tests.

Principal coordinates analysis (PCoA) combined with non-parametric multivariate variance (PERMANOVA) on Bray-Curtis distances were performed to visualize the relationships between samples. Additionally, Bray–Curtis dissimilarity analysis was employed to calculate the dissimilarities between samples (Bray and Curtis 1957). The fungal taxonomy and relative abundance at phylum and genus level were calculated by UNITE database. The feature importance analysis based on ASVs was conducted using the “randomForest” package (Breiman 2001). Ranked lists of ASVs biomarkers in order of Random Forests reported feature importance scores were achieved over 100 iterations of the algorithm. The identification of biomarker taxa was conducted through 10-fold cross-validation with five repetitions. The “vegan” package was used to screen for the combination of soil properties that exhibited the highest correlation with significant features (Oksanen et al. 2019). The “psych” package was used to analyze the correlation and significance between biomarker taxa and soil properties (William Revelle 2023). For each soil variable, multiple linear regression (MLR) analysis and variance decomposition analysis were conducted using the lm() function and “relaimpo” package (Grömping 2006), resulting in the determination of the importance of soil variable compared to fungal biomarker taxa.

## Results

### Effects of different treatments on soil properties and enzyme activities of American ginseng continuous cropping obstacle

**Fig. 1:**
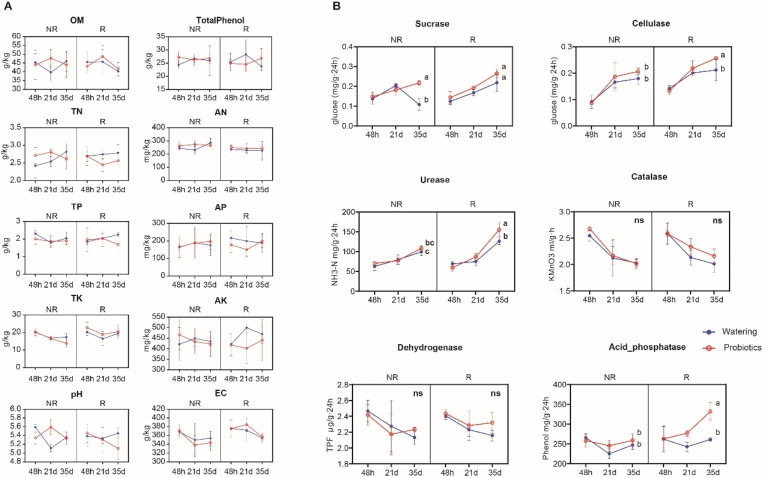
Comparison of soil properties and enzyme activities under rotation and probiotic *Bacillus* treatments. **(A)** The mean (± SD) values of soil properties. **(B)** The mean (± SD) values of soil enzyme activities. ANOVA and Tukey’s multiple comparison tests for each time point, and the statistical significances (p < 0.05) were indicated by different letters

To track the effects of different treatments on American ginseng continuous cropping obstacle soil, we detected soil properties and enzyme activities at 48 h, 21 days and 35 days. There were no significant differences in soil properties and soil total phenolics among different treatments (Fig. 1A). On the other hand, rotation and probiotic *Bacillus* treatment had significantly affects on the activities of some soil enzymes. Compared with NR_W group, rotation groups (R groups) significantly increased the sucrase and urease activities. Probiotic treatment groups (P groups) significantly increased cellulase, sucrase and acid phosphatase activities. Sucrase, cellulase, urease and acid phosphatase activities were significantly increased with the rotation and probiotic *Bacillus* treatment (R_P group) (Fig. 1B).

### Rotation affected the alpha diversity of soil fungal community in continuous cropping obstacle over time-series

**Fig. 2:**
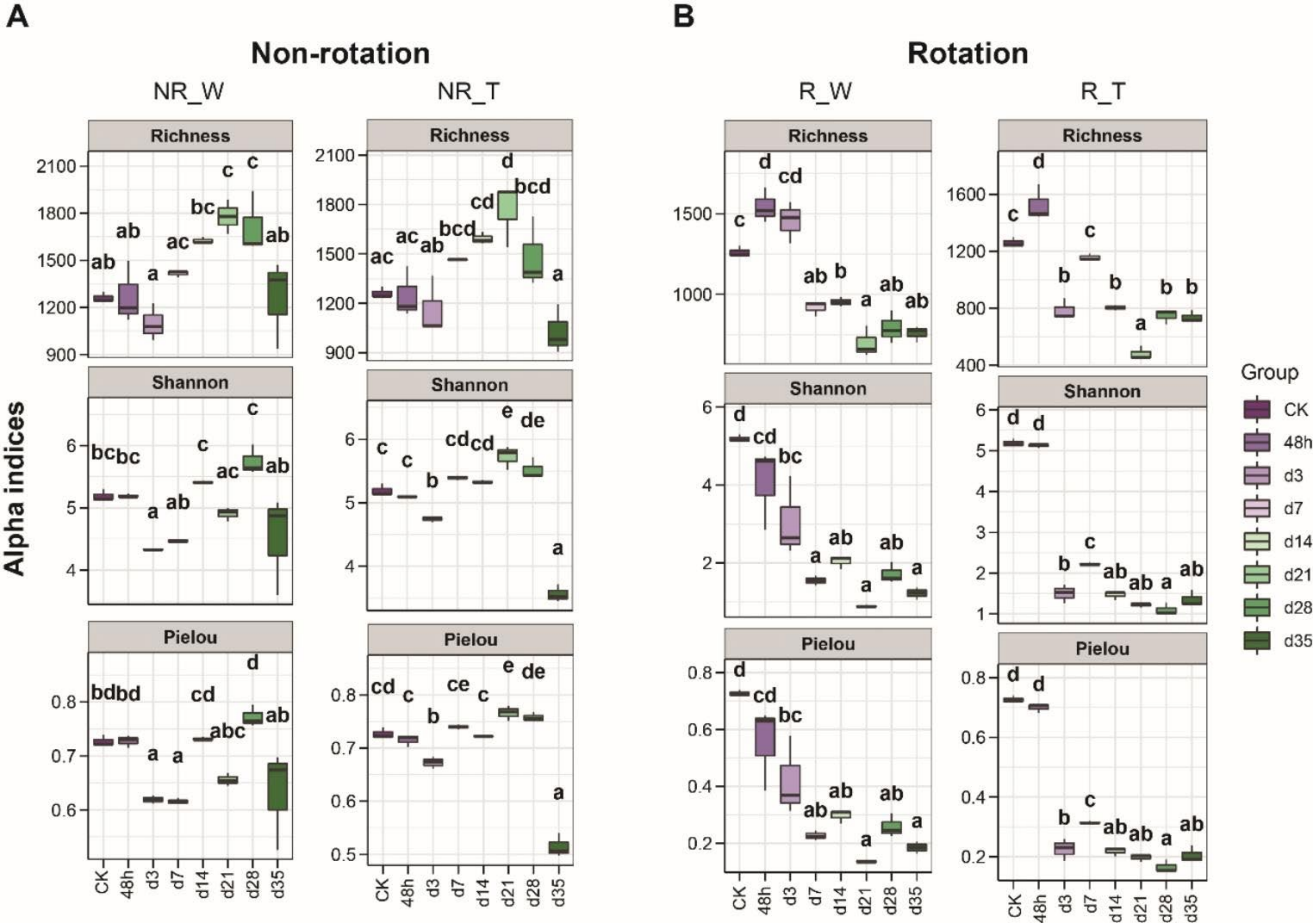
Comparison of fungal diversity (Richness and Shannon alpha diversity indices) and evenness (Shannon and Pielou alpha diversity indices) under rotation and probiotic *Bacillus* treatments based on ASVs. ANOVA and Tukey’s multiple comparison tests for each time point, and the statistical significances (p < 0.05) were indicated by different letters

To determine the changes of soil fungal alpha diversity in different treatments, the Richness, Shannon, and Pielou alpha diversity indices based on ASVs were evaluated. We found that rotation treatments affected alpha diversity in the fungi community of American ginseng soil significantly. With or without probiotic *Bacillus*, the richness, diversity and evenness of fungi community decreased significantly with time under rotation treatment (Fig. 2A). In the Non-rotation groups, the alpha diversity of the fungal community first increased then decreased significantly from 48 h to day 35 (Fig. 2B). Notably, we found that the probiotic *Bacillus* treatment (NR_P and R_P group) had no significant effects on the soil fungal community alpha diversity (Fig. 2).

### Changes of fungal community structure with time under different treatments

**Fig. 3:**
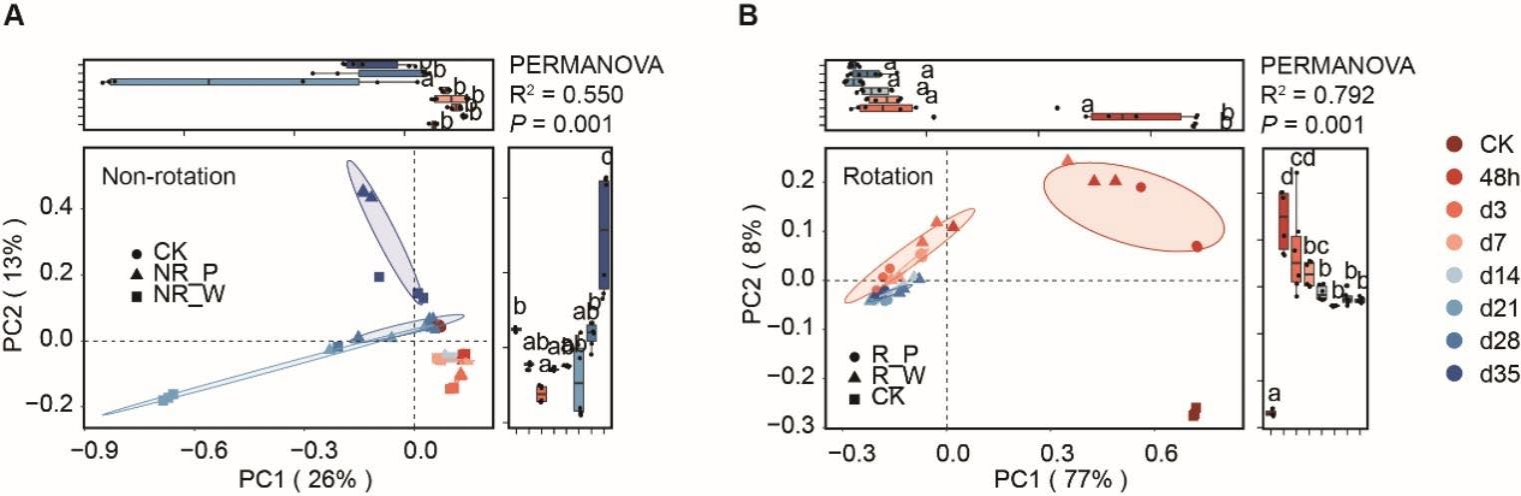
Comparison of soil fungal community structure at different sampling time points based on ASVs (PCoA; pairwise comparisons based on PERMANOVA). **(A)** Non-rotation treatment **(B)** rotation treatment. Successive colors represent sampling time points, and different shapes represent different treatments

In order to elucidate the effect of different treatments on the structure of fungal community in soil with continuous cropping obstacle, the Principal coordinate analysis (PCoA) method based on Bray-Curtis dissimilarity matrix was performed to detected the change trend of fungal community structure over time. In Non-rotation treatment groups, the fungal community structure was significantly different (PERMANOVA, R^2^ = 0.550, *P* = 0.001) with time, and the percentages of variation explained by PC1 and PC2 axis were 26 and 13%, respectively. Bray–Curtis distance revealed that the soil fungal community formed two distinct clusters, which separated along the PC2 axis, indicating that the largest source of variation in the soil fungal communities was treatment time. In particular, we found that soil fungal community structure was significantly different before and after the 14th day of rotation treatment (Fig. 3A).

Furthermore, rotation treatment had a significant effect on the soil fungal community. (variation explained by PC1 and PC2 axis were 77 and 8%). The Bray-Curtis distance of rotation groups was separated at the early stage of treatment time (PERMANOVA, R^2^ = 0.792, *P* = 0.001), and the distance was unchanged after 14 days. The fungal microbiota distance variations between watering (R_W group) and probiotics *Bacillus* treatment (R_P group) shortened or even overlapped over time-series, suggesting that the effects of probiotics on soil fungal community structure will weaken over time (Fig. 3B).

**Fig. 4:**
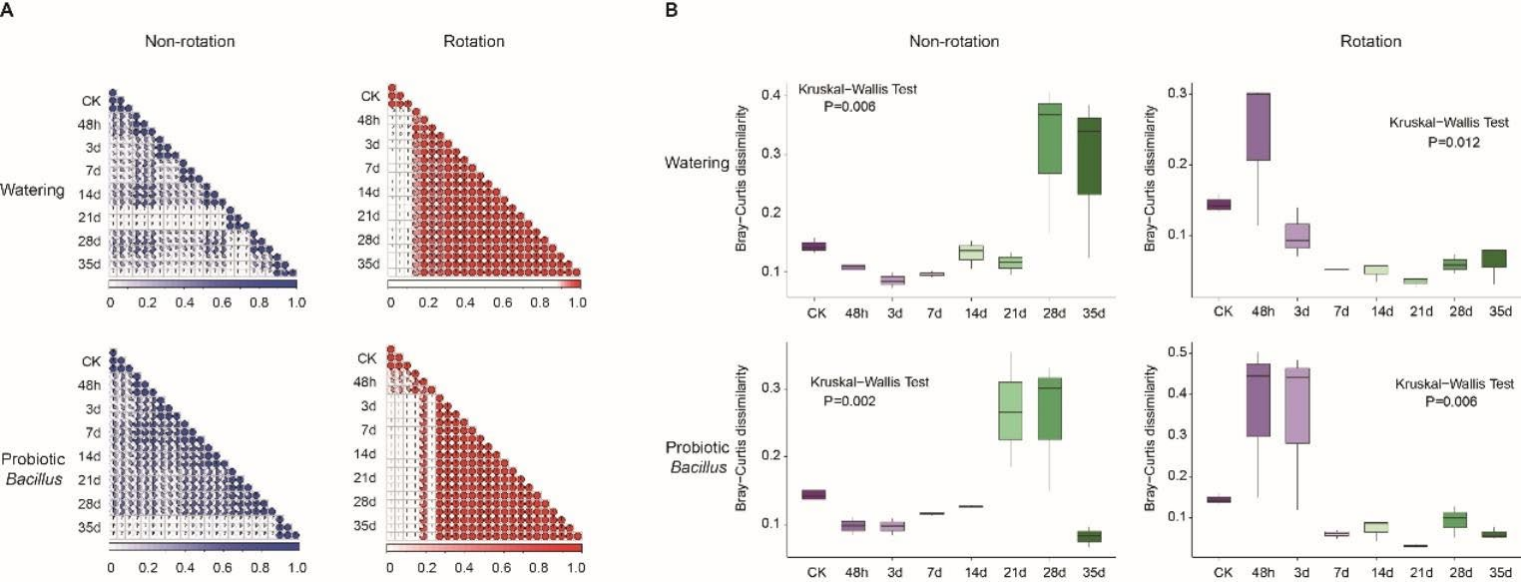
Correlation and variation of fungal community structures over time-series in different treatments. **(A)** Correlation of fungal communities with treatments time. The degree of correlation was highlighted in color–the stronger the correlation, the darker the color. **(B)** Bray-Curtis dissimilarity of fungal community over treatments time. Kruskal-Wallis test for each time point

Furthermore, we focused on the correlation of fungal communities over time-series in each treatment. Compared with the NR_W group, the soil fungal community correlation was more disturbed by the rotation treatment (red color). The fungal community formed a stable structure within 48 h to 7 days and persisted until the end of the experiment. Also, we found that probiotic treatment (R_P group) could delay the formation of soil fungal community stability. Interestingly, watering cycle also appeared to have a lasting effect on the soil fungal community (Fig. 4A).

Patterns of Bray-Curtis dissimilarity in fungal communities differed significantly under different treatments over time-series. Rotation treatments showed significantly changes in the fungal community dissimilarity at 48 h (*P* = 0.012 and *P* = 0.006). And the probiotic *Bacillus* treatment increased the duration of dissimilarity up to 3 days. In the NR_P group, the dissimilarity of fungal community significantly increased from day 21 to day 28 after treatment with probiotic *Bacillus* (*P* = 0.002). Also, we found that watering treatment also significantly affected the fungal community dissimilarity after 28 days (*P* = 0.006) (Fig. 4B).

### Soil fungal community composition in American ginseng continuous cropping obstacle under different treatments

**Fig. 5:**
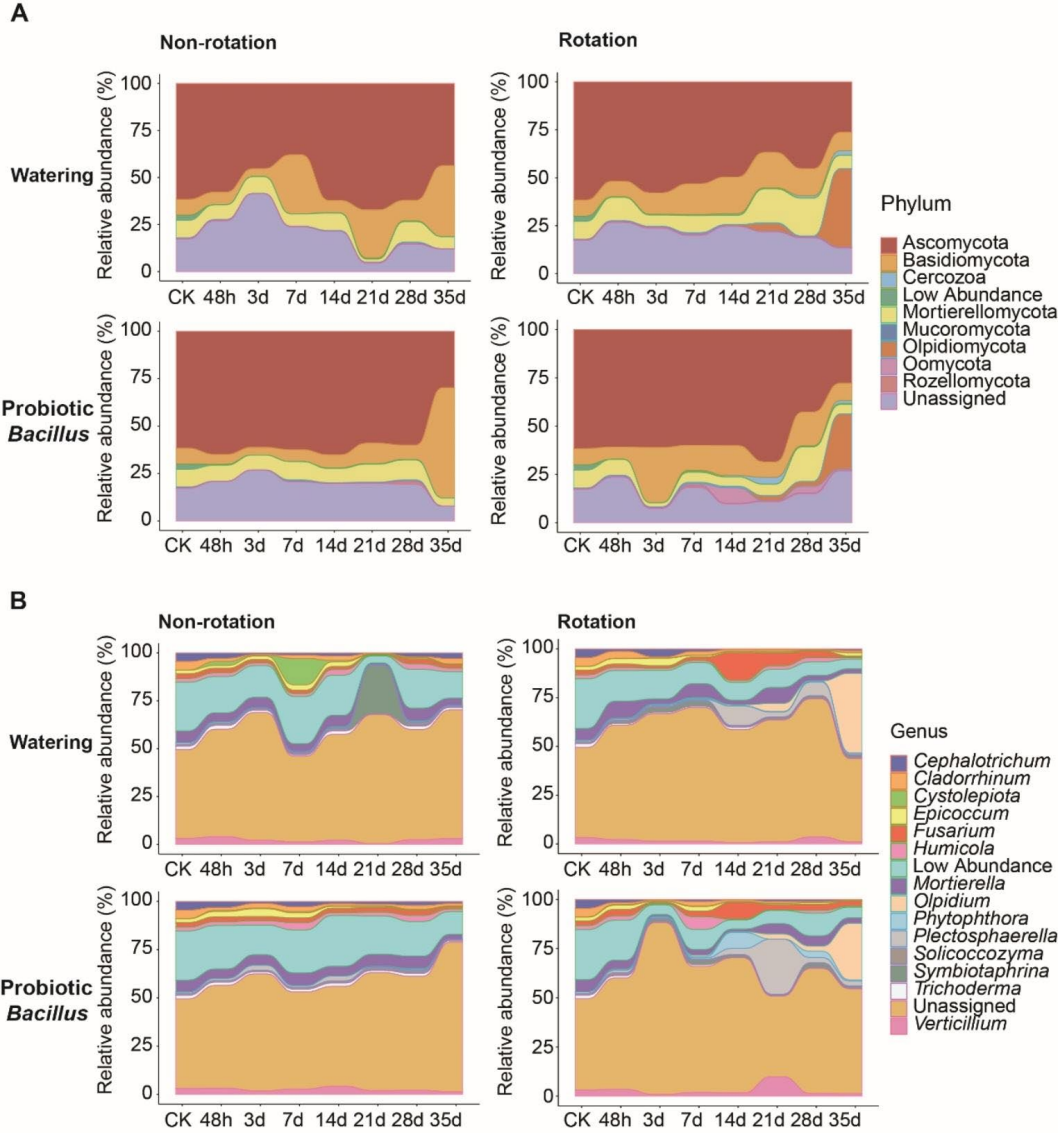
The succession of fungal species relative abundance over time-series at the level of the top 10 phyla and the top 15 genera. **(A)** Top 10 phyla of soil fungal taxa in American ginseng continuous cropping obstacle. **(B)** Top 15 genera of soil fungal taxa in American ginseng continuous cropping obstacle

The succession of the relative abundance of fungal communities over time-series was investigated at the level of the top 10 phyla and the top 15 genera. The main fungal phyla were Ascomycota, Basidiomycota, Mortierellomycota and Olpidiomycota. In non-rotation treatment groups, the relative abundance of Ascomycota and Basidiomycota fluctuated noticeably over time. The relative abundance of Ascomycota decreased while Rozellomycota increased after 28 day with rotation treatment (Fig. 5A).

At genus level, the fungal community consisted mainly of *Fusarium*, *Cephalotrichum*, *Cladorrhinum*, *Epicoccum*, *Mortierella* and *Olpidium*. For the rotation treatment groups, the relative abundance of *Fusarium* and *Olpidium* increased after 7 day. Also, we found that the relative abundance of *Plectosphaerella* and *Verticillium* increased at 21 day with probiotic *Bacillus* treatment. In non-rotation treatment groups, there was little variation in the relative abundance of species. Interestingly, the relative abundance of *Cystolepiota* and *Solicoccozyma* were increased at 7 day and 21 day in NR_W group, respectively (Fig. 5B).

### Fungal biomarker taxa in continuous cropping obstacle soil under different treatments and their correlation with soil properties

To determine the fungal biomarker taxa, we regressed the relative abundance of fungi at the genus level among the treatments at different sampling time points using the random forest algorithm, to establish a series of models to correlate fungal microbiota composition with different treatments. The models explained 83.0%, 84.9%, 83.0% and 70.8% of the fungal microbiota variance related to respective treatment. The number of fungal biomarker taxa was 7, 7, 15 and 15 in NR_W, NR_P, R_W, and R_P group though 10-fold cross-validation with five repeats, respectively. In order of time-discriminatory importance, the fungal biomarker taxa across treatments time were shown in Fig. 5. The results showed that crop rotation was an important factor affecting the distribution of soil fungal biomarkers of American ginseng continuous cropping obstacle. At the same time biomarker species were enriched at different treatment times. *Ceratobasidium*, *Hannaella*, *Epicoccum* and other genus were enriched on 14 d of NR_W treatment. While *Epicoccum* and *Hannaella* showed relatively high abundance from 48 h to 7 d in NR_P treatment. The number of fungal biomarkers was higher in rotation soils compared to non-rotation treatments (7 vs. 15), and was enriched at 48 h. With rotation treatments, all fungal biomarkers were accumulated at 48 h and decreased during treatment time, which contained the *Cladorrhinum*, *Oidiodendron*, *Mariannaea* and other beneficial fungi. In addition, all biomarkers were negatively correlated with hydrolases (such as cellulase, sucrase and urease), and positively correlated with oxidoreductases (such as dehydrogenase and catalase) and acid phosphatase. The fungal biomarker taxa of rotation treatment groups were significantly correlated with more soil properties.

**Fig. 6:**
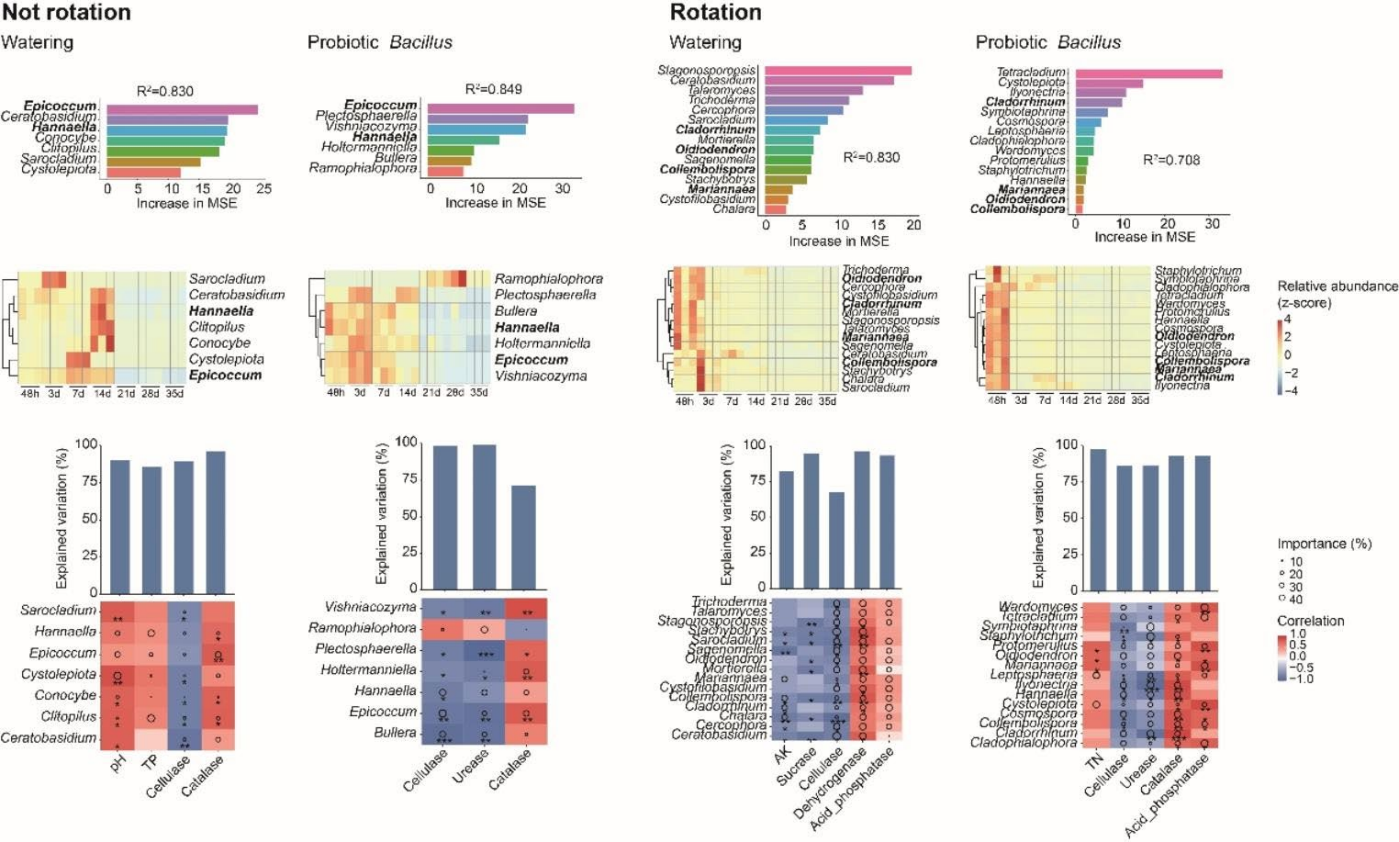
Biomarkers, relative abundances and their correlations with soil properties of American ginseng continuous cropping obstacle in different treatments. The association between fungal genera and treatments time was identified in different treatments using the Random Forests method. The number of genera in the model with the lowest error rate was selected through cross-validation, and the genera were sorted in descending order of contribution. Heatmaps showed time-related relative abundance of biomarkers. The histogram and heatmap showed the correlation, importance, and explanation degree of the most relevant soil properties and the biomarkers in the respective treatment

## Discussion

### Fungal community dynamics and soil enzyme activities changes during rotation and probiotic treatments

Previous studies showed that plantation patterns could change the physicochemical properties and microbial communities of American ginseng soil. However, these studies were conducted at single or sparse points in time, and little attention had been paid to changes in fungal communities over short periods (Jiao et al. 2019b). We conducted a short-term crop rotation and probiotic cross-experimental treatment and intensive sampling on American ginseng continuous cropping obstacle soil. The results demonstrated that the fungal community in the American ginseng continuous cropping obstacle soil under different treatments changed dynamically over time, and the fluctuation of the fungal community was more severe under the rotation treatment, which is consistent with previous research (Zhang et al. 2020a). Probiotic *Bacillus* treatments, on the other hand, had minor effect on soil fungal community, whether rotation or not (Fig. 2). These results suggested that plant roots may recruit various taxa of fungi even if they were not detected in the initial continuous cropping obstacle soil.

For example, *Fusarium* and *Plectosphaerella* begun to accumulate in the soil after 7 days of rotation and maintain a high level in the middle and late stages of the rotation (Fig. 5B). The trend was strong in both the rotation and probiotic *Bacillus* treatment, although they differed in terms of relative abundance and duration of enrichment. Previous studies also found that potential pathogenic fungi Sordariomycetes accumulated significantly in the old ginseng fields with American ginseng (Ji et al. 2021b). These results were indicated that plant root system may be a selective environment for potential pathogenic microorganisms. Previous study was also found that fungal pathogens like *Fusarium* was more abundant in soil of diseased American ginseng plants (Ji et al. 2021a), indicating that potential fungal pathogens may begin to accumulate in the early stage of plants root growth. In addition, we observed that soil enzyme activities were more responsive to probiotic *Bacillus* treatment, which significantly increased the activities of soil sucrase, cellulase, urease and acid phosphatase from 21 to 35 days (Fig. 1). This finding was consistent with that of previous studies of *Bacillus* (Ramesh et al. 2011; Rajper et al. 2016). Therefore, rotation and probiotic treatment formed the different succession of soil fungal community over time-series in American ginseng continuous cropping obstacle.

### Rotation treatment changed the recruiting pattern of soil fungal microbiota

Soil fungal microbiota in rotation treatment were significantly different from CK group and gradually shifted at later time points (Figs. 2B and 3B), indicating that a rapid aggregation of fungi occurred during the first 48 h. These results had been demonstrated in root bacterial microbiota that communities started to change dramatically at the beginning of rice planting (Edwards et al. 2018; Zhang et al. 2018). Besides, our results revealed that the fungal structure changed dramatically in 48 h to 3 d under the rotation treatment, while it changed significantly during 28 d to 35 d under the non-rotation treatment, which might be due to the directional enrichment of soil fungi by plant roots (Schlatter et al. 2020). Interestingly, probiotic treatment did not seem to strongly affect the enrichment process of soil fungal community, even when probiotics were initially introduced to the soil (Fig. 4). This may be because the inoculated *Bacillus* does not promote the growth of indigenous bacteria in soil, resulting in inability to combined action together to achieve disease suppression (Tao et al. 2020).

Compared to probiotic *Bacillus*, rotation treatment mainly formed the different succession of soil fungal community over time in American ginseng continuous cropping obstacle. Basidiomycota were the one of most dominant phylum in American ginseng soil (Ji et al. 2021b). In our study, Basidiomycota was dramatically decreased in rotation treatment and increased in non-rotation. Olpidiomycota was abundantly enriched at the late stage of rotation treatment. Previous studies showed that *Olpidium* belonging to Olpidiomycota could accumulate in rhizosphere, and most species could break down cellulose and chitin (Lay et al. 2018; Illescas et al. 2020). Our results suggested that plant roots had the ability to recruit *Olpidium* in the continuous cropping obstacle soil of American ginseng (Fig. 5). We also observed that plant growth had an enrichment effect on *Fusarium*, which was reported mainly in the study of rhizosphere pathogenic fungi of American ginseng (Westerveld et al. 2023). These results suggested that dormant potentially beneficial and pathogenic fungi might be enriched through rhizosphere selection of plants.

### Correlation of fungal biomarker taxa with soil properties with different treatments over time-series

We identified fungal biomarker genus of different treatments correlating with time by using a Random Forests model. As expected, the fungal biomarkers of different treatments had specific clustering patterns, respectively. With non-rotation treatment, the enrichment time sequence was different in NR_W and NR_P groups. Most biomarkers in NR_W group, such as *Ceratobasidium*, *Hannaella*, *Epicoccum* and other genus, were enriched on 14 d. While in NR_P group most biomarkers like *Epicoccum* and *Hannaella* showed relatively high abundance from 48 h to 7 d (Fig. 6). Studies found that as an endophytic fungus of *Panax ginseng*, *Epicoccum* could produce numerous biologically active compounds to promote the growth of ginseng root (Wang et al. 2020; Zhang et al. 2023a). Mycorrhizal Fungus *Ceratobasidium* contributed to the accumulation of flavonoids in rhizosphere plants (Zhang et al. 2020b). And *Hannaella* was reported to have good biocontrol potential (Zhao et al. 2022). Our results also showed that only *Ramophialophora* was enriched at 28 d. As saprophytic fungi, *Ramophialophora* was reported to improve soil nutrition, regulate soil structure and inhibit soil-borne diseases (Clocchiatti et al. 2020; Wu et al. 2022). Most biomarkers were significantly negatively correlated with soil cellulase and positively correlated with catalase. While the correlation between *Ramophialophora* and soil enzymes was opposite to that of other biomarkers, indicating that *Ramophialophora*, as a potential beneficial fungus in soil, exhibited different growth and enrichment patterns compared with other biomarkers under the pressure of *Bacillus* introduction. The root microenvironment, as a means of nutrient exchange and information transfer, can affect the accumulation and growth of microbiota (Saleem et al. 2018). With rotation treatments, beneficial fungal biomarkers, such as *Cladorrhinum*, *Oidiodendron*, and *Mariannaea*, which had positive effects on plant growth and soil health, were accumulated at 48 h (Yu et al. 2022; Ali et al. 2022; Yin et al. 2022). All biomarkers were negatively correlated with hydrolases, and positively correlated with oxidoreductases and acid phosphatase, indicating that the decrease of biomarkers with rotation treatment within 35 d might be related to the increase of soil sucrase, cellulase, urease and acid phosphatase, while the decrease of catalase and dehydrogenase. In summary, the study revealed the enrichment in soil potentially beneficial fungal biomarkers and soil-borne pathogenic fungi over time-series under short-term crop rotation and probiotics treatments. The continuous succession mechanism of soil fungi community in American ginseng continuous cropping obstacle have yet to be monitored over the long-term.

## Conclusions

Our current investigation elucidated that rotation and probiotic *Bacillus* treatments formed the different succession of soil fungal community over time-series in American ginseng continuous cropping obstacle. First, rotation and probiotic *Bacillus* treatments significantly increased the sucrase, cellulase, urease and acid phosphatase activities in soil. Second, compared to probiotic *Bacillus*, rotation treatment significantly reduced the fungal Richness, Shannon, and Pielou indices from 48 h to day 35, and began to significantly affect the Beta diversity distance and dissimilarity degree at 48 h. Furthermore, the rotation treatment significantly affected the variation of fungal taxa. Beneficial fungal biomarkers like *Cladorrhinum*, *Oidiodendron*, and *Mariannaea* were accumulated within 48 h, whereas pathogenic biomarkers such as *Fusarium* were enriched after day 3. Finally, the fungal biomarkers were negatively correlated with cellulase, sucrase and urease, and positively correlated with dehydrogenase, catalase and acid phosphatase. Therefore, our study revealed the succession of fungal communities over time-series during the initial stage of rotation and probiotic *Bacillus* treatments. However, the specific mechanisms by which crop rotation and probiotics impacts on the available nutrients and fungal communities in American ginseng continuous cropping obstacle soil needs further research.

## Data Availability

The raw data from 31 samples were available from the Sequence Read Archive (SRA) under the accession number: PRJNA954137.
